# Unusual Symptoms of Lacertus Syndrome: A Case Report

**DOI:** 10.3390/jcm14030685

**Published:** 2025-01-22

**Authors:** Bartosz Chlebanowski, Paweł Walkowiak, Emilia Czupryniak, Marcin Domżalski, Justyna Pigońska

**Affiliations:** 1 Department of Orthopedics, University Clinical Hospital No. 2, 90-549 Łódź, Poland; bartek.chlebanowski@gmail.com (B.C.);; 2 Sporto Ltd., 90-038 Łódź, Poland; 3 Department of Neurology with Stroke Subdivision, John Paul II Regional Hospital of Belchatow, 97-400 Belchatow, Poland; walkowiak.pawe@gmail.com; 4 Medical Faculty, Medical University of Łódź, 90-419 Łódź, Poland; emczupryniak@gmail.com; 5 Department of Extrapyramidal Disorders, Central Clinical Hospital Medical University of Lodz, 92-213 Łódź, Poland

**Keywords:** Lacertus syndrome, electroneurography, HRUS, median nerve

## Abstract

**Background:** Lacertus syndrome, a condition characterized by median nerve compression at the elbow due to anatomical variations, is often misdiagnosed. This case report describes a 53-year-old female patient who presented with severe lateral elbow and shoulder pain, previously diagnosed with cervicalgia and tennis elbow. **Methods:** Upon admission, she reported significant pain (NRS—Numerical Rating Scale 8/10) and occasional radiating paresthesia in the first three digits of her left hand. Clinical examination revealed weakness in the wrist and finger flexion, which was exacerbated by a positive Cutaneous Scratch Test (CST), while Magnetic Resonance Imaging (MRI) of the cervical spine showed no definitive abnormalities. Electroneurography (ENG) indicated reduced amplitude in the left anterior interosseous nerve. Ultrasound excluded carpal tunnel syndrome but identified nerve flattening beneath the pronator teres muscle. A surgical decompression of the median nerve was performed, resulting in immediate improvement in muscle strength and significant pain reduction (NRS 1/10) postoperatively. Follow-up evaluations confirmed substantial recovery in nerve function and morphology. **Conclusions:** This case illustrates the atypical presentation of Lacertus syndrome, emphasizing the need for comprehensive diagnostic approaches that include clinical, imaging, and neurophysiological assessments. Awareness of this syndrome is crucial for differential diagnosis in patients exhibiting uncharacteristic symptoms, such as shoulder or elbow pain, to ensure timely and effective treatment.

## 1. Introduction

The median nerve originates from C 6,7,8 and the Th1 root and is formed from the lateral and medial fascicle and next from the lower part of the brachial plexus. The most common cause of the median lesion is typical carpal syndrome in the wrist. Less frequently, but typically underdiagnosed, is the lesion of the median nerve distally to the elbow joint at the level of the lacertus aponeurosis. Diagnostic challenges arise due to the lack of standardized tools for examination and, potentially, also the dynamic nature of the condition. We present a case of Lacertus Syndrome in a patient who, in addition to the typical symptoms, exhibited nonspecific signs which were misdiagnosed as cervicalgia and brachialgia for an extended period. The thorough clinical examination—including dynamic assessment, which included a neurodynamic test, along with expanded electrophysiology and high-resolution ultrasound (HRUS)—led to the correct diagnosis and the qualification for surgery [1]. Consequently, the patient demonstrated rapid clinical improvement which was also reflected in the HRUS and Electroneurography (ENG) findings.

## 2. Detailed Case Description

A 53-year-old female patient without comorbidities was admitted to hospital with severe pain localized in the lateral part of the left elbow and shoulder. She had a prior diagnosis of cervicalgia and tennis elbow syndrome. Upon admission, the patient reported a severe pain assessed as 8/10 on the Numerical Rating Scale (NRS) and occasional, rare radiating paresthesia affecting the first, second, and third digits of the left hand.

### 2.1. Clinical Examination and Initial Findings

**Figure 1:**
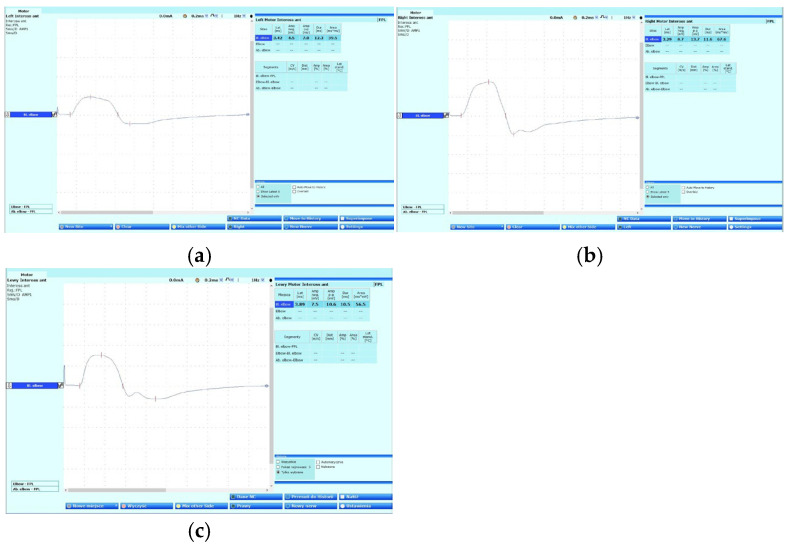
Anterior interosseous nerve—electroneurography images recorded: (**a**) left limb before surgery on 20 February 2024, (**b**) right limb on 20 February 2024, (**c**) left limb after surgery on 11 March 2024—with the improvement of the amplitude.

Physical examination revealed no significant abnormalities, except for a positive Cutaneous Scratch Test (CST) described by Hagert et al. [2,3]. Comparison of the wrist and finger flexion strength before and after CST showed a significant reduction in strength on the MRC scale from 5 to 4 points [4]. Moreover, the test provoked numbness in the distal phalanges of the first three digits after compression of the median nerve [3]. Furthermore, neurodynamic examination of the left median nerve revealed paresthesias along the course of the median nerve with radiation to Fingers 1–3, although the neurodynamic examination for radial and ulnar nerve was negative [5]. Magnetic resonance imaging (MRI) of the cervical spine showed no definitive abnormalities suggestive of radiculopathy. Radiographic evaluation of the elbow and electroneurography (ENG), performed prior to admission, did not demonstrate any abnormalities, effectively excluding carpal tunnel syndrome. However, the ENG revealed a notable reduction in the amplitude of the left anterior interosseous nerve (recorded from the flexor pollicis longus muscle, according to Felice’s method [6] when compared to the asymptomatic side (Figure 1)).

**Figure 2:**
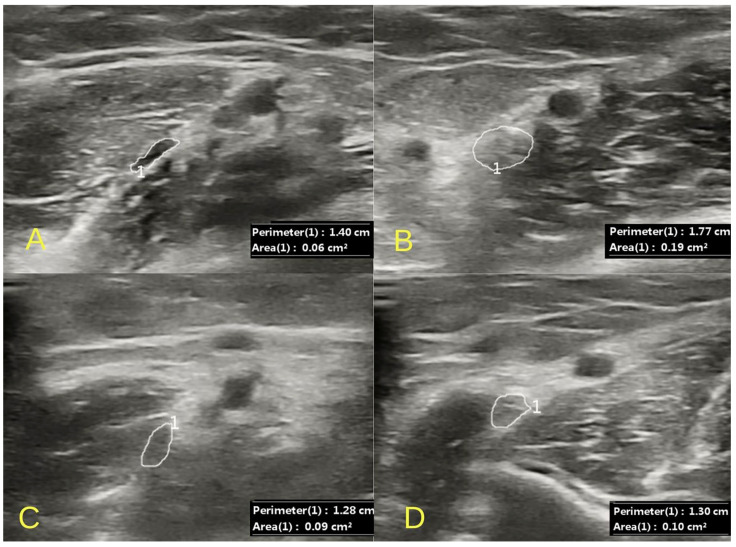
An ultrasound image of the massive compression of the median nerve (1) between the heads of the pronator teres muscle (**A**) with the swelling of the nerve at the level of the lacertus fibrosus (**B**). Postoperative evaluation reveals a normal median nerve both at the site of the previously noted compression (**C**) and in the ulnar fossa (**D**). Images were obtained with the D7L40L linear probe of the Chison CBit4.

HRUS of the left median nerve was performed. No ultrasonographic features of carpal tunnel syndrome were found. At the level of the wrist, the median nerve was visualized with a preserved echostructure, showing no signs of compression and demonstrating normal mobility within the carpal canal on dynamic examination of the second finger. On the forearm, massive compression of the median nerve was observed within the heads of the pronator teres muscle. Secondary edema with the obliteration of the echogenic structure of the median nerve in the ulnar fossa, under the lacertus fibrosus, was also visualized. In correlation with the ultrasound image, there was increased pain in the area of compression and the swelling of the median nerve upon palpation with the ultrasound probe. A comparative evaluation was performed with the contralateral asymptomatic side, where no features of compression in the pronator teres muscle and edema in the ulnar fossa were visualized (Figure 2).

### 2.2. Surgical Intervention and Outcome

**Figure 3:**
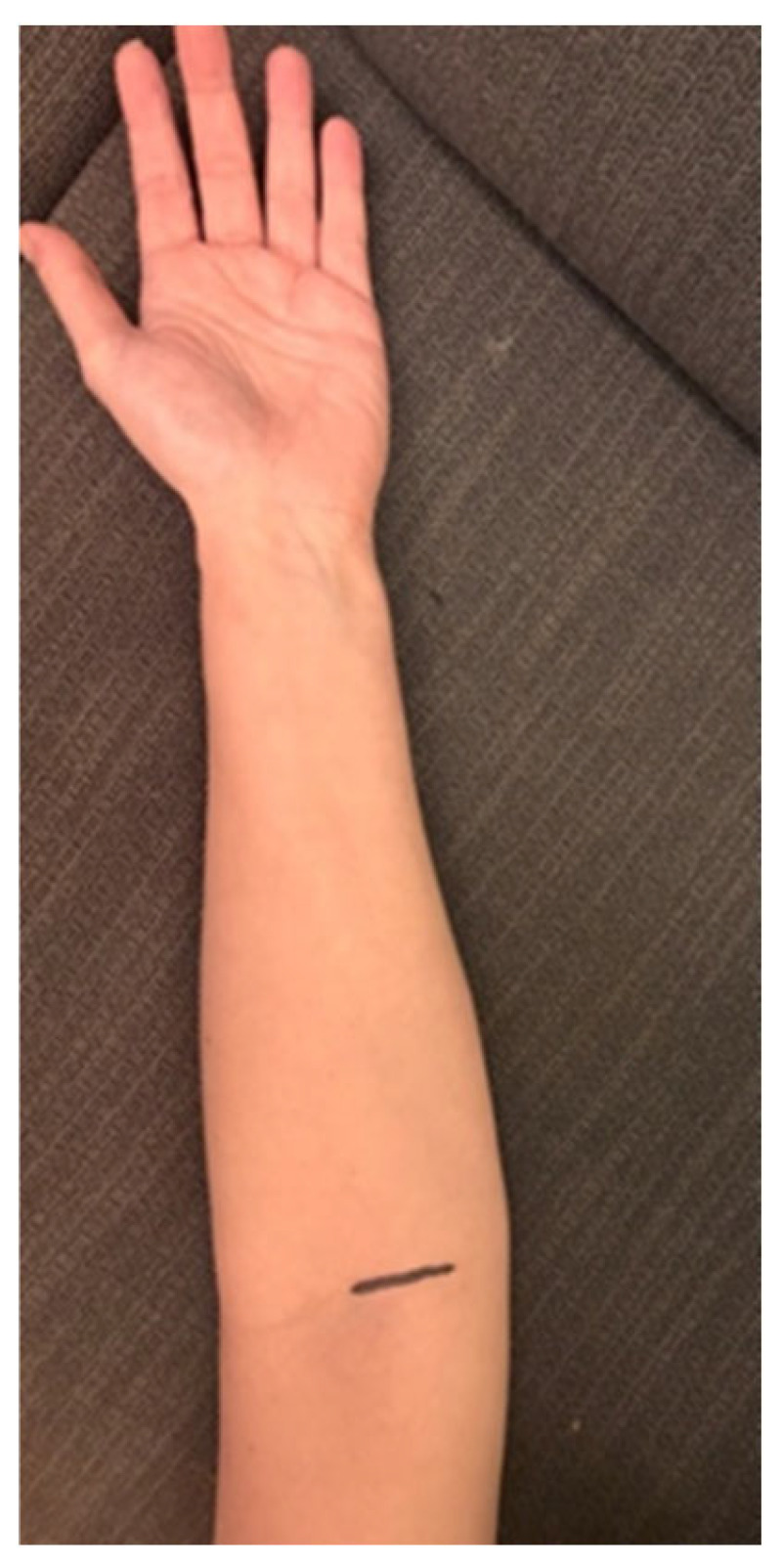
Marked surgical access.

**Figure 4:**
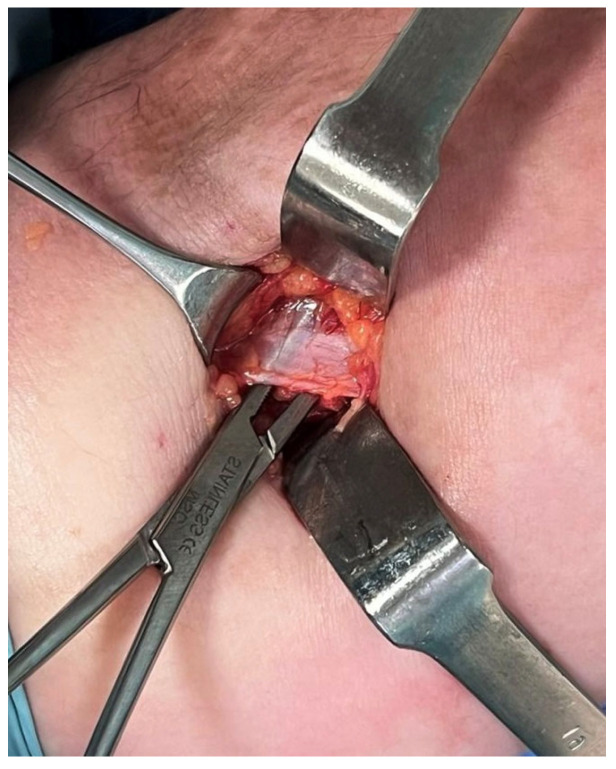
Lacertus fibrosis intraoperation photography.

Conservative treatment in the form of intensive physiotherapy and neuromobilization had no apparent effect [7]. Additionally, an injection of corticosteroids and lidocaine was administered, which provided short-term pain relief [8]. Subsequently, the patient underwent surgical decompression of the median nerve under WALANT anesthesia [9]. The mini-open surgical procedure was conducted with the skin incision made medial to the biceps tendon (Figure 3). The pronator teres muscle was mobilized and the lacertus fibrosis was intersected under direct visualization [10] (Figure 4). Intraoperative observations revealed immediate improvement in muscle strength and the morphology of the nerve visibly changed. Postoperatively, the patient experienced a rapid reduction in pain, with their NRS score decreasing to 1/10 within hours.

Follow-up assessments conducted one week post-surgery included ENG and ultrasound, both of which demonstrated significant improvement. The ENG showed an increase in the amplitude of the anterior interosseous nerve, and ultrasound confirmed normalization of the nerve morphology.

## 3. Discussion

The compression of the median nerve between the two heads of the pronator teres muscle was first described by Seyffarth in 1951 [11]. Anatomical studies have demonstrated that the structures contributing to median nerve compression at the elbow are associated with thick aponeurosis originating from the two heads of the pronator teres and the flexor muscles of the wrist and fingers. The tension in this aponeurosis is dynamic, depending on elbow movements and pronation, distinguishing this condition from a typical tunnel syndrome [2,12]. Instead, it represents a dynamic compression syndrome influenced by the functional activity of the elbow. The unusual symptom in our case was pain in the lateral side of the elbow, likely caused by the compression of the sensor branch of the median nerve which supports the elbow joint. Therefore, differential diagnosis was made between cervical radiculopathy and lateral epicondylitis of humerus. The clinical presentation of Lacertus Syndrome commonly includes weakness in the flexor muscles of the wrist and fingers, firstly affecting the first, second, and third digits. These symptoms are typically exacerbated by provocation, such as the scratch test performed over the fibrosis area. Patients may also report paresthesia, described as periodic radiation to the first three digits, and radiating pain in the forearm. In our case, severe pain localized at the elbow was noted, likely resulting from the compression of the median nerve branch, which innervates the elbow joint. This hypothesis is supported by the complete resolution of symptoms following surgical intervention. Muscle weakness observed in this condition is especially manifested during activities requiring fine motor control, such as brushing teeth, combing hair, or holding a phone. Electroneurography and electromyography studies often fail to reveal abnormalities; however, in our evaluation, we detected axonal injury of the anterior interosseous nerve, recorded via the flexor pollicis longus, with rapid postoperative improvement. In this case, conservative treatment was unsuccessful, and due to severe pain and the patient’s expectation of a rapid recovery, surgical treatment was pursued. However, some studies show that conservative treatment can be effective. Patients are advised to avoid provocative positions such as elbow flexion, pronation of the forearm, and forceful griping [8]. Furthermore, physical therapy methods that incorporate kinesiotaping, including nerve gliding, are especially advantageous, as they assist in preserving the nerve’s mobility within its sheath, which may alleviate symptoms associated with nerve entrapment. Additionally, the administration of oral anti-inflammatory medications can offer symptomatic relief during the early stages of management [1].

Currently, imaging methods—such as magnetic resonance imaging and physical examination allow for highly effective diagnosis of root syndromes. Neurophysiological examination, especially electromyography, is recommended when symptoms are ambiguous or unclear, especially in cases where MRI reveals features that do not correlate with the patient’s clinical condition. In contrast, a normal MRI image and the absence of clinical abnormalities often render electromyography unnecessary, as it is considered an invasive test. In such cases, greater emphasis is placed on the diagnosis of peripheral nerve conditions using HRUS [13]. Ultrasonography also plays an important role in diagnosis, particularly due to the diverse anatomy of peripheral nerves [14,15].

Nowadays, ultrasound examination plays an important role in the diagnosis of mono- and polyneuropathy [16]. It is also valuable in diagnosing this syndrome, as it enables the dynamic visualization of median nerve deformation beneath the aponeurosis and the subsequent improvement after surgical decompression. Despite these advances, the role of additional diagnostic tests remains limited due to the lack of standardized methods. Occasionally, ENG may show a reduction in conduction velocity in the distal part of the median nerve, but statistical data on this finding are lacking. Future research should aim to establish standardized diagnostic criteria and further explore the pathophysiology of this condition. In cases with atypical symptoms, such as pain in the shoulder or elbow, Lacertus Syndrome should be considered in the differential diagnosis.

## 4. Conclusions

This case highlights the potential for rapid clinical and electrophysiological improvement following surgical intervention for median nerve compression at the elbow. These findings underscore the importance of integrating clinical, imaging, and neurophysiological assessments in the diagnosis and management of similar cases.

## Data Availability

The data are contained within the article.
